# Functional outcome following proximal humeral interlocking system plating for displaced proximal humeral fractures

**DOI:** 10.4103/0973-6042.59971

**Published:** 2009

**Authors:** David S. Thyagarajan, Samarth J. Haridas, Denise Jones, Colin Dent, Richard Evans, Rhys Williams

**Affiliations:** Department of Trauma and Orthopaedics, University Hospital of Wales, Cardiff, South Wales

**Keywords:** Proximal humeral fractures, locking plate fro proximal humeral fractures, Philos plating

## Abstract

**Aim:**

To assess the functional outcome following internal fixation with the PHILOS (proximal humeral interlocking system) for displaced proximal humeral fractures.

**Patients and Methods:**

We reviewed 30 consecutive patients treated surgically with the proximal humeral locking plate for a displaced proximal humeral fracture. Functional outcome was determined using the American Shoulder and Elbow Society (ASES) score and Constant Murley score.

**Results:**

Average age of the patients was 58 years (range, 19-92 years). The average overall ASES score was 66.5. The average overall Constant score was 57.5.

**Conclusion:**

Our results show that good fracture stability was achieved, and the functional outcome was very good in younger patients and it declined with increasing age. Early mobilization of the shoulder can be achieved without compromising fracture union.

## INTRODUCTION

We present this study of 30 patients with displaced proximal humeral fractures treated with PHILOS plate. Their functional outcome was assessed using the ASES (American Shoulder and Elbow Society) score and Constant Murley scoring system.

Surgical management of proximal humeral fractures is rarely indicated as they are seldom displaced or angulated. It is estimated that only 20% of proximal humeral fractures require surgical treatment.[1] The indication for fixing such a fracture depends on the fracture pattern, quality of bone and the age and activity of the patient.[2] The goal is to achieve near-anatomical reduction and stabilization so as to achieve early mobilization. We report the outcome following this new technique as a surgical option in the management of proximal humeral fractures. We hypothesize that the locking plates provide good fracture stability and they help to advocate early mobilization without compromising fracture union.

## PATIENTS AND METHODS

We treated 30 consecutive patients who had displaced proximal humeral fractures, with PHILOS (proximal humeral interlocking system) plating at a single large teaching hospital. The fracture was classified using Neer's classification. The classification is based on the degree of displacement and angulations of the anatomical segments regardless of the level of fracture or the mechanism of injury.[3] The criteria used to select these patients for surgery were the amount of displacement of the fracture fragments (45 degrees of angulation and 1 cm of displacement) and the quality of function of the shoulder preoperatively. The operations were carried out by 2 specialist shoulder surgeons. The mean age of the 30 patients was 58 years (range, 19-92 years). Only 5 patients were younger than 35 years; 8 were between 36 and 55 years; 9 were between 56 and 75 years; 8 were between 76 and 95 years. The cause of injury was mainly a simple fall, but other causes were road-traffic accidents, skiing and fall from a ladder. The surgery was carried out within 10 days of the injury in 17 patients and within 2 weeks in the other 13 patients. Postoperatively the patients were assessed clinically and radiologically. The average follow-up period was 9 months (range, 4-12 months). Functional shoulder assessment was done using the ASES[4] score and Constant scoring system.[5]

### Physiotherapy regime

Our physiotherapy regime consisted of polysling for 3 weeks with pendulum exercises, followed by active assisted external rotation to neutral and active assisted flexion. At 6 weeks they were allowed full range of movements.

**Figure 1:**
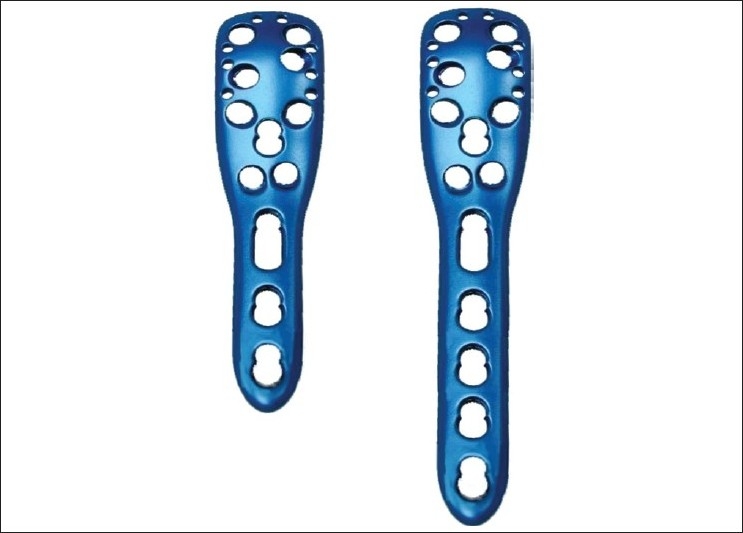
Diagram of the proximal humeral interlocking system^®^ plate (× 2) showing 9 locking compression plate screw holes and 10 suture holes proximally and 1 long hole with 3 or 5 combi holes distally

**Figure 2:**
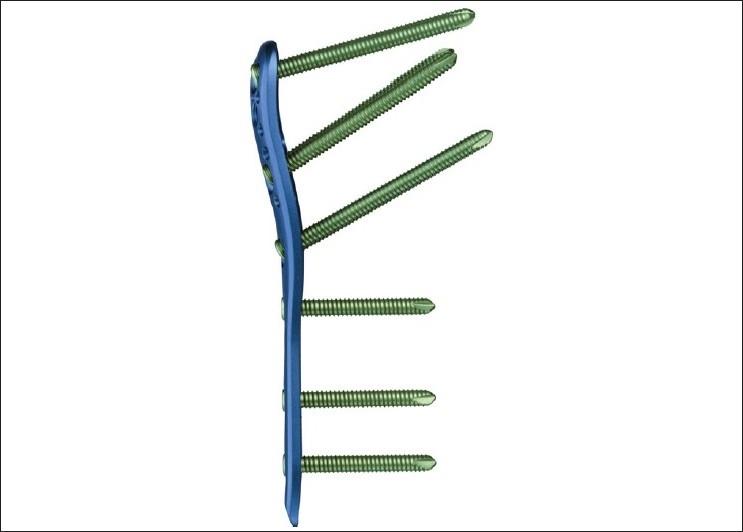
Diagram showing different screw angles

System description: The elements of the PHILOS plate are shown in Figures 1 and 2.

### Surgical technique

Through a deltopectoral approach [Figure 3a], the fracture site is exposed taking care of the soft tissue envelope to maintain a good vascular supply [Figure 3b]. The anterolateral branch of the anterior humeral circumflex artery, which is the primary blood supply to the proximal humerus, can be damaged while exposing the tendon in the bicipital groove, and care should be taken to avoid this complication as this may jeopardize the blood supply to the humeral head and increase the risk of avascular necrosis.[6] The fracture is then reduced anatomically, and the locking plate is applied onto the proximal humerus. The technically demanding part of the operation is to get the correct version of the humerus while applying the plate. The height of the implant is set by inserting the guide wire [Figure 3c]. It cannot be too high due to risk of impingement, and it cannot be too low as there will be insufficient holes to put the screws into the head of humerus [Figures 3d, 4a, b and 5a, b].

**Figure 3a:**
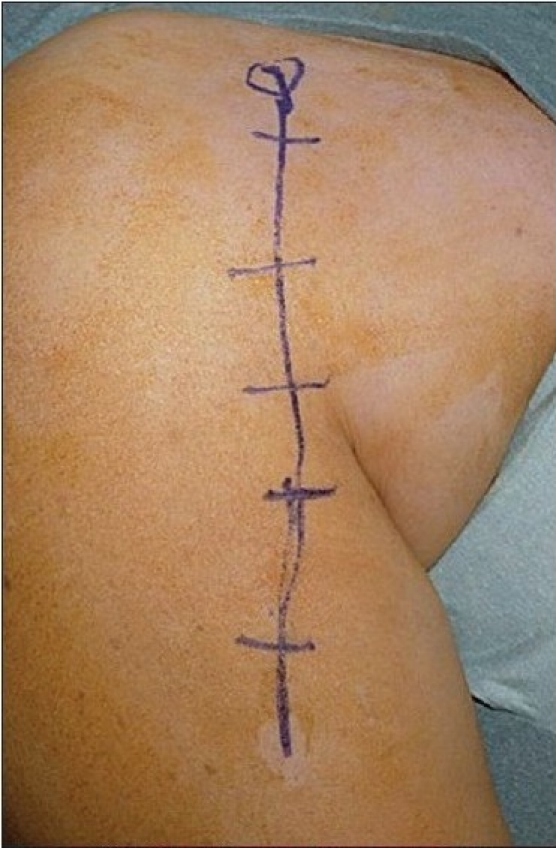
Surgical appearance of the extensile deltopectoral incision used for the proximal humeral interlocking system plate

**Figure 3b:**
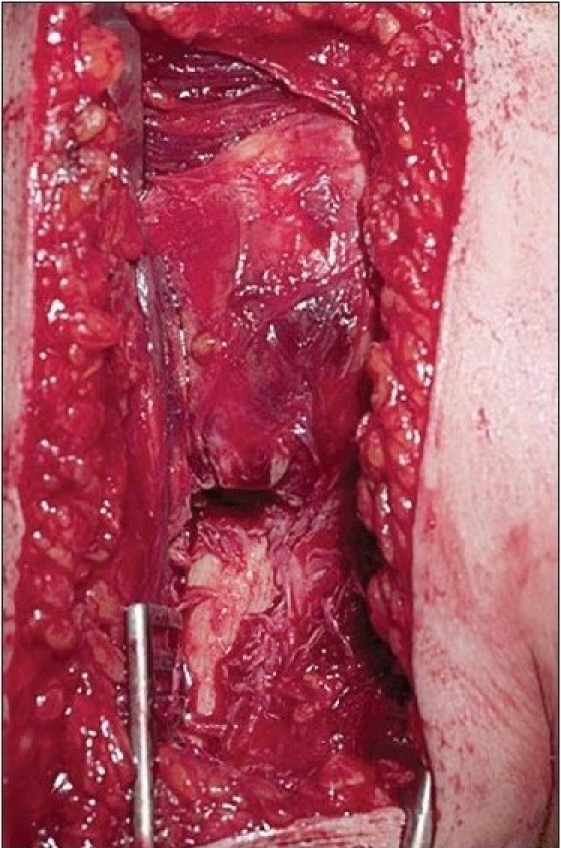
The fracture site exposed without disturbance of the soft tissue envelope

**Figure 3c:**
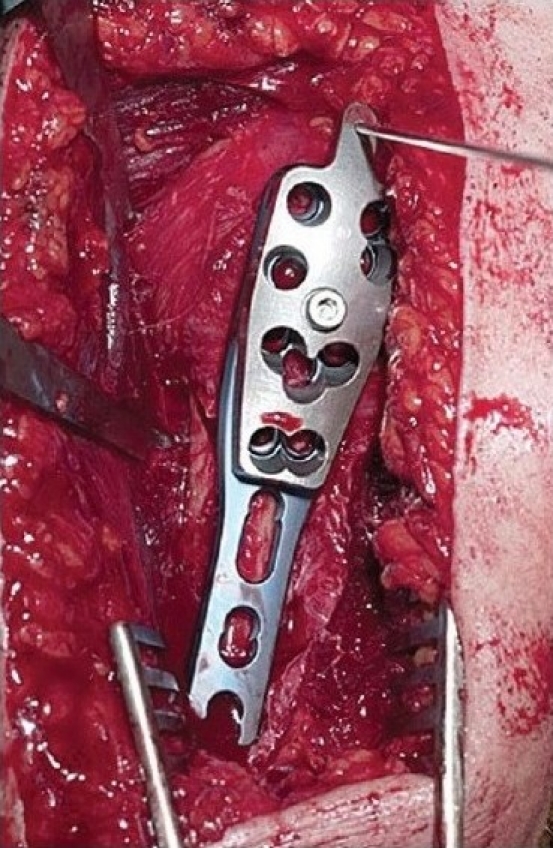
Insertion of the guide wire to set the implant at the exact position needed

**Figure 3d:**
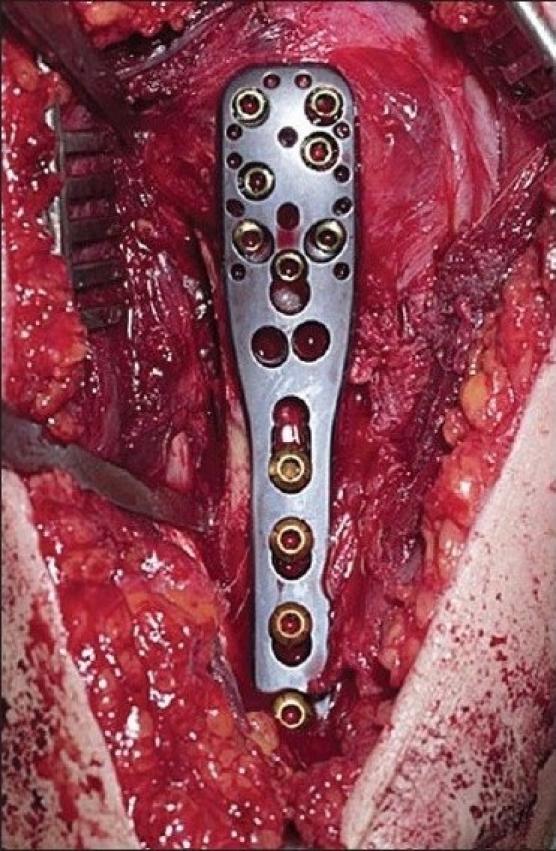
The end result of the surgery using proximal humeral interlocking system^®^ plate with the unicortical screws at the articular surface and bicortical screws at the shaft

**Figure 4a:**
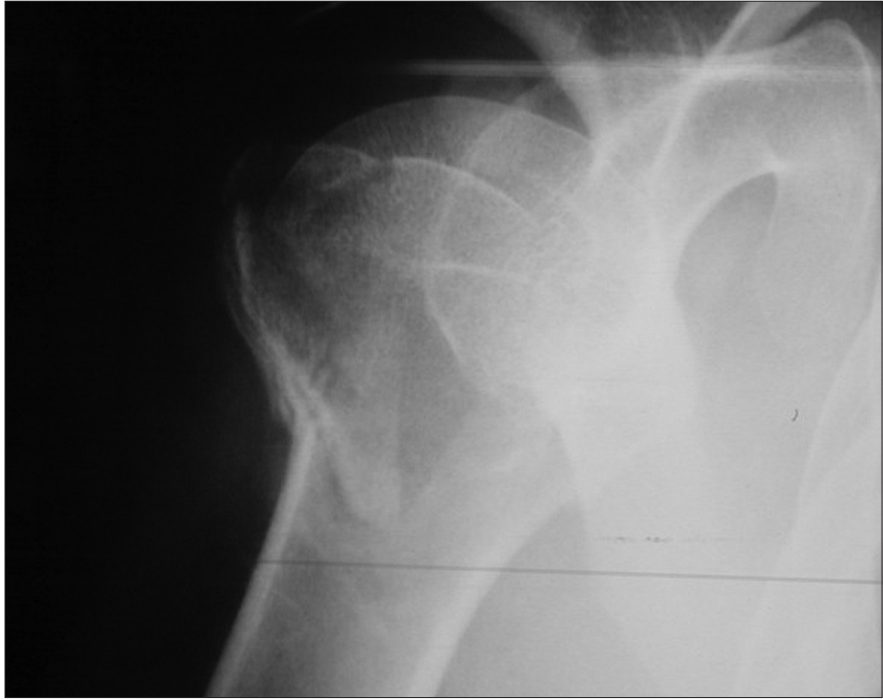
The radiographic appearance of the three-part fracture of the proximal humerus

**Figure 4b:**
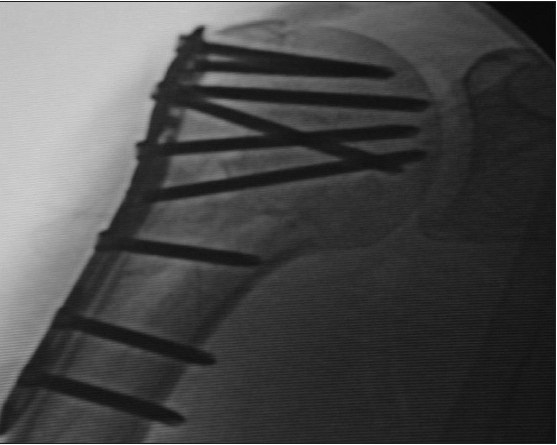
Same three-part fracture fixed with proximal humeral interlocking system plate

**Figure 5a:**
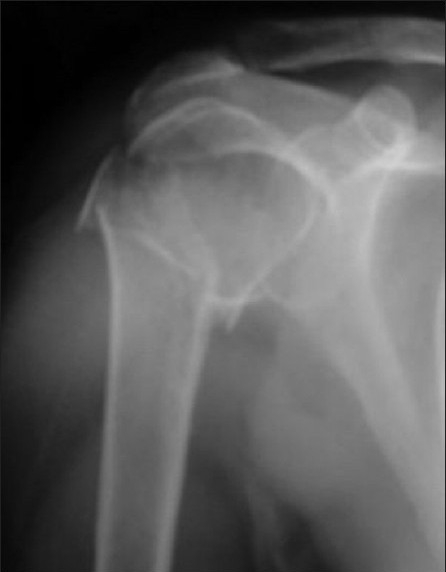
The radiographic appearance of the four-part fracture of the proximal humerus

**Figure 5b:**
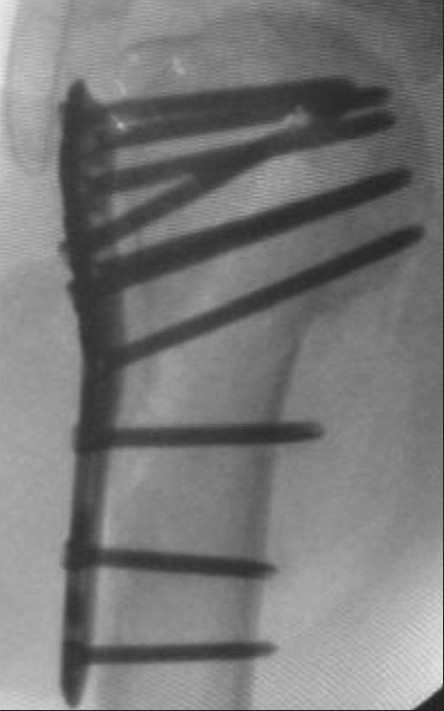
Same four-part fracture fixed with proximal humeral interlocking system plate

## RESULTS

Of the 30 patients who underwent surgery with proximal humeral locking plate, we reviewed 29 patients as 1 patient had died due to coexisting medical ailments. Table 1 shows the characteristics of the 29 patients included in this study in relation to their age and the outcome score. Out of the 29 patients, 19 were females and 10 were males. There were 6 two-part, 14 three-part and 10 four-part fractures. Radiological union was achieved within 12 weeks following the surgery. The average overall ASES score was 66.5. The average overall Constant score was 57.5. See Table 2‐5 for details.

One patient developed wound infection, which was initially treated with wound debridement, washout and antibiotics, but subsequently she required a removal of implant and a revision of locking plate once the infection had settled. Two patients developed signs of subacromial impingement; these patients belonged to the earlier subgroup of patients in the series where the implant was positioned too far cranially. Hence the implants were removed; the fractures had united at the time of implant removal.

**Table 1:** Age groups of patients and averages of the American Shoulder and Elbow Society and Constant Scores, respectively

Age	Total no. of patients (*n* = 29)	ASES score	Constant score
15-35	5	82/100	72/100
36-55	7	70/100	71/100
56-75	9	59/100	53/100
76-95	8	55/100	35/100

ASES: American Shoulder and Elbow Society

**Table 2:** American shoulder and elbow society objective assessments (average)

Age	Forward elevation % range of movements achieved	External rotation at 0°	External rotation at 90° abduction	Internal rotation	Strength
15-35	85-95	55°-64°	55°-64°	Level of T12	19/20
36-55	85-95	35°-44°	55°-64°	Level of L3	14/20
56-75	65-74	35°-44°	35°-44°	Level of sacrum	13/20
76-95	55-64	35°-44°	25°-34°	Level of sacrum	10/20

**Table 3:** American Shoulder and Elbow Society subjective assessments (average)

Age	Pain score 0 = no pain	Activity of daily living, e.g., put on a coat, comb hair, toileting, etc.
15-35	4/10	26/30
36-55	5/10	22/30
56-75	7/10	19/30
76-95	8/10	16/30

**Table 4:** Constant objective assessments (average)

Age	Forward elevation and lateral elevation % ROM achieved	External rotation	Internal rotation	Power
15-35	85-95	Hand on top of the head, elbow back	Level of T12	15/25
36-55	85-95	Hand on top of the head, elbow forward	Level of L3	15/25
56-75	65-74	Hand on top of the head, elbow forward	Level of sacrum	10/25
76-95	55-64	Hand behind head, elbow held back	Level of sacrum	5/25

**Table 5:** Constant subjective assessments (average)

Age	Pain score 15 = no pain	Work/recreation/sleep	Positioning of the shoulder
15-35	12/15	9/10	Neck to top head
36-55	14/15	9/10	Above head
56-75	10/15	7/10	Neck to top head
76-95	8/15	6/10	Xiphoid to neck

## DISCUSSION

The majority of proximal humeral fractures are treated conservatively. There are different surgical options for the fixation of proximal humeral fractures, e.g., interfragmentary fixation with sutures,[7,8] percutaneous pinning,[9] intramedullary fixation[10] and hemiarthroplasties.[11,12] The recent trend is to use less invasive procedures for reduction and fixation of the fracture.[13,14] The lesser invasive the procedure the more are the operative prerequisites, viz., 1) good bone stock, 2) minimal comminution of the tuberosity, 3) patient willing to participate in postoperative physiotherapy regimes and 4) advanced operative skills.[2]

Fixation of proximal humeral fractures with plates and screws has been associated with complications such as pullout of screws in osteoporotic bone, subacromial impingement and avascular necrosis of the humeral head due to excessive periosteal stripping.[15,16] Kristiansen and Christensen have reported a high incidence of fixation failure following use of T-buttress plates in fixation of proximal humeral fractures. Wijgman *et al*. have reported good intermediate and long-term results in 87% of patients who had three-and four-part fractures fixed with T-buttress plate. The average age of the patients in their study was 48 years.[17]

More recently newer implants such as the plan tan humerus fixator plate, Polaris nail and the PHILOS plate have been used for fixation of proximal humeral fractures. The plan tan humerus fixator plate involves placing 2 cancellous compression screws in the humeral head together with a plate on the humeral shaft. Although most authors have reported satisfactory results in young patients, there have been high rates of complications and fixation failures in elderly patients with osteoporotic bone. Sadowski *et al*. have reported a 100% failure rate with the use of this device in elderly patients.[18] The use of Polaris nail has shown some favorable results in younger and older patients with two-part fracture.[19]

This study has presented a new surgical option in the management of displaced proximal humeral fractures. It combines the principles of fixation with a conventional plate with those of locking screws. The plate is pre-shaped and contoured for the proximal humerus. The benefits of this implant are that it gives enhanced purchase in osteopenic bone, there is no loss of reduction or varus/valgus angulations, the locking screws into the plate provide angular and axial stability of the construct and it is a low-profile plate. We have been able to produce the early results with regard to functional outcome following use of locking plates (PHILOS). The only technically demanding part of the operation is to obtain the correct version of the humerus for accurate plate positioning. We therefore encountered some impingement problems with the earlier subgroup of patients as it was a relatively new implant and a new technique being used. With this plate, there is less insult to the vascular supply of the fracture as the soft tissue envelope is not disturbed and hence there is less chance of osteonecrosis. The other demanding aspect is to avoid placing the plate too proximally on the humerus with resulting impingement of the top of the plate on the acromion. This can be avoided by using a K wire inserted through a hole at the top of the plate, which should line up with the tip of the greater tuberosity. This is done during initial positioning of the plate. Positioning the plate too high can also lead to incorrect placement of the divergent screws in the humeral head. Care should be taken to avoid penetration of the head and subsequent chondrolysis with proximal interlocking screws. Image intensifier is necessary to check correct positioning and placement of the implant and screws, respectively.

From the results of the functional outcome, it is clear that this procedure gives a good functional score in young patients. The elderly were able to return to independent active living. The ASES scores revealed that 55% to 64% of the forward elevation and external rotation was achieved on active range of motion. The most difficult movement for the older subgroup was internal rotation, which was up to the sacrum. With regard to the activities of daily living, the elderly group scored 16/30 with ASES system and 6/10 with the constant scoring system.

In elderly patients, osteopenic bone in combination with a thin and/or ruptured rotator cuff predisposes to unpredictable clinical results. In our study, the results of surgical fixation in the elderly age group were not so good. The overall ASES and Constant scores for the age group 76-95 years were only 55/100 and 35/100, respectively.

There are limitations in our study. Firstly the total number of patients in the study was small, and only 8 out of 29 patients were in the age group 76-95 years. Secondly, few of the elderly patients had persistent pain and stiffness despite radiological union. Poor rehabilitation potential following surgery could be a possible explanation.

However, more recently Koukakis *et al*. published a series of 20 patients with two-, three-and four-part fractures treated with this plate and have shown no difference in functional outcome between younger (<65 years) and older (>65 years).[20]

We achieved good fracture fixation with no implant failures even in the osteopenic bones. We believe that the locking plate provides good fracture stability and allows early mobilization of the shoulder without compromising fracture union. We would recommend the use of the PHILOS plate as a surgical option in the management of displaced proximal humeral fractures.
